# Supplementary material for: Computational analysis of hygromorphic self-shaping wood gridshell structures
Source: R Soc Open Sci. 2020 Jul 1;7(7):192210. doi: 10.1098/rsos.192210 (PMC7428239; doi:10.1098/rsos.192210)
Supplement: S2–S3 [file rsos192210supp2.pdf]

# Supplementary material

## Computational analysis of hygromorphic self-shaping wood gridshell structures

Philippe Grönquist[1,2,3], Prijanthy Panchadcharam[2], Dylan Wood[4], Achim Menges[4], Markus Rüggeberg[1,2] and Falk K. Wittel[2]

[1]Laboratory for Cellulose & Wood Materials, Empa, 8600 Dübendorf, Switzerland
[2]Institute for Building Materials, ETH Zurich, 8093 Zürich, Switzerland
[3]Institute of Structural Engineering, ETH Zurich, 8093 Zürich, Switzerland
[4]Institute for Computational Design and Construction, University of Stuttgart, 70174 Stuttgart, Germany

*Movie S1: Caption:* : **Exemplary gridshell self-shaping process in computational and physical model**. FE simulation of self-shaping process of a 5x5 gridshell made out of beech wood and physical reconstruction. U3: Out of plane displacements in mm. Parameters: Strip geometry: 600 mm length, 12 mm width, 2 mm passive layer thickness (radial orientation), 5 mm active layer thickness (longitudinal orientation). Boundary conditions: Change in moisture content from 14% to 21%, physical sample relocation from 65% to 95% relative humidity climate at 20°C.

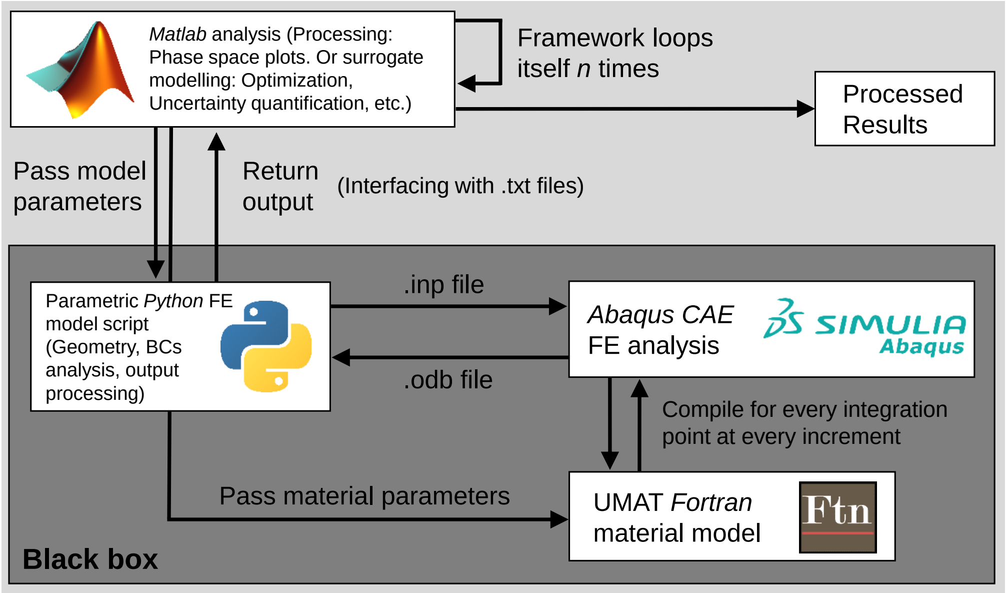


Figure S2: **Framework for parametric FE analysis.** Analysis parameters such as FE model geometry or boundary conditions (BCs) can be automatically sampled via *Matlab* scripts and passed for FE analysis to parametric *Python* scripts. FE model material parameters can be directly passed to user material subroutines (UMAT) written in *Fortran 77* (not used in this study, displayed for the sake of interest). The framework is based on a black box approach and, therefore, facilitates parametric analyses such as phase diagrams, optimizations, or uncertainty quantification, which can be conducted in frameworks decoupled from the FE analyses. (*The authors state not be be associated or sponsored by any of the organizations whose logo is appearing in this figure*).

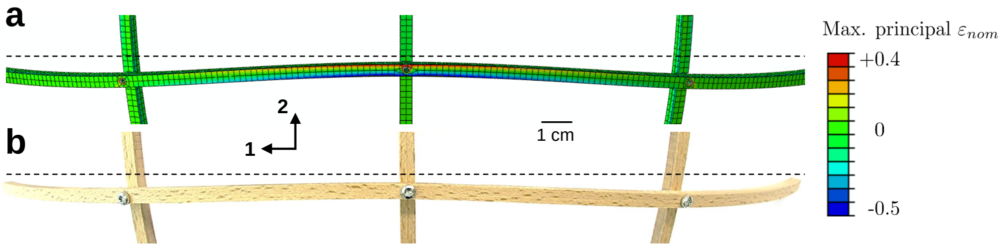


Figure S3: **In-plane bending of a border strip from configuration A visualized in the 1-2 plane.** **a**: Maximal principal nominal strains of deformed FE model configuration. It can be recognized that at the crossing between middle and border strips, the in-plane bending strain takes over the self-shaping (out of plane) strains as the maximal principal strain components of the strain tensor. **b**: Image of Experimental configuration A.
