# Supplementary material for: Computational analysis of hygromorphic self-shaping wood gridshell structures
Source: R Soc Open Sci. 2020 Jul 1;7(7):192210. doi: 10.1098/rsos.192210 (PMC7428239; doi:10.1098/rsos.192210)
Supplement: S4 [file rsos192210supp3.pdf]

```matlab
% #############################################################
% Description
% #############################################################

% This function runs a parametric Finite Element Method Analysis (Abaqus
% 6.14) of wood bilayer gridshells in dependence of number of bilayer
% strips, length, thicknesses of active and passive layers, and width. The
% results are stored as raw data (displacements) in the matrix C. The
% displacements are further processed to curvatures along the x- and
% y-directions of the gridshell system

% Works with:
%   Microsoft Windows 10 Enterprise
%   Matlab R2016b
%   Abaqus/CAE 6.14-1

% Author:
% Philippe Grönquist (pgroenquist@ethz.ch), August 2019

% Initialize Matlab
clc; clear; close all;


% #############################################################
% Define the parameters
% #############################################################

% Define size of parameter space
Size = 11;

% Fixed parameters
n = 6;      % Number of strips in total, n/2 must be an odd number
L = 500;    % Bilayer length in mm

% Vaiable parameters
h1 = linspace(1,5,Size);    % Thickness of passive layer
h2 = linspace(4,20,Size);   % Thickness of active layer
w  = linspace(5,45,Size);   % Width of bilayer strip


% #############################################################
% Run analysis
% #############################################################

% Create a 4D matrix, according to size of results matrix (RES),
% this needs to be adapted manually in terms of the fixed parameters and FE model setup in Python
C = zeros(Size,Size,18,4);

% Loop
for i = 1:Size
    for j = 1:Size

        % Re-arrange variables in a single vector
        X(1) = n;
        X(2) = L;
        X(3) = w(i);
        X(4) = h1(j);
        X(5) = h2(j);

```

```
        try

            % Call the FE execution function
            RES = Gridshell_Analysis(X);

            % Store raw results matrix of FE analysis in a 4D matrix C
            C(i,j,:,:) = RES;


        catch Me

            % If error during FE analysis, fill containers with zeros
            C(i,j,:,:) = zeros(18,4);

            % Print warning message
            disp('########################################');
            disp(['ERROR for i = ',num2str(i),', and j = ',num2str(j)]);
            disp('########################################');

        end
    end
end


% #############################################################
% Calculate curvatures (for 6x6 Gridshell)
% #############################################################

% Natural curvatures (from single strip FE model) in [1/m]
kk0 = [4.0368, 2.8834, 2.2427, 1.8349, 1.5526, 1.3456, 1.1873, 1.0623, 0.9611, 0.8776, 0.8074];

for i = 1:Size
    for j = 1:Size

            % Access results of 4D matrix
            RES = squeeze(C(i,j,:,:));

            % call curvature calculation function
            [Ax,By,Cxy] = Gridshell_Curvature(RES,L);% in 1/mm

            % curvatures along directions x and y
            Kx(i,j) = Ax*1000;% in 1/m
            Ky(i,j) = By*1000;% in 1/m

            % Gaussian curvature
            K(i,j)  = Cxy*1000*1000;% in 1/m2

            % Dimensionless curvature by thickness
            h = (h1(j)+h2(j))./1000;% in m
            Kdl(i,j) = K(i,j).*h^2;

            % make a matrix of natural curvatures
            k0(i,j) = kk0(j);

            % Dimensionless curvature by natural curvature
            Kdl(i,j) = K(i,j)./k0(i,j);

    end
end
```

```
% (manuscript figure)
% Plot the results by using "imagesc(X)" where X is Kx, Ky, K, k0, Kdl, etc.


% ##############################################################
% Results (RAW DATA, NOT PART OF THIS SCRIPT)
% ##############################################################

% Gridshell curvature along x in [1/m]
Kx =

  Columns 1 through 6

    3.2150    2.7978    2.3233    1.9642    1.6825    1.4722
    2.5765    2.4441    2.1795    1.8962    1.6574    1.4566
    2.0974    2.2070    2.0371    1.8265    1.6220    1.4274
    1.6125    1.9787    1.9310    1.7772    1.5873    1.4167
    0.9150    1.7683    1.8058    1.7234    1.5565    1.4061
    0.5771    1.6250    1.6960    1.6598    1.5369    1.3968
    0.4141    1.5338    1.6171    1.5962    1.5115    1.3845
    3.3261    1.4526    1.5522    1.5636    1.4785    1.3717
    3.4062    1.4442    1.5248    1.5311    1.4518    1.3590
    3.4313    1.4357    1.4712    1.4790    1.4269    1.3414
    3.4426    1.3997    1.4440    1.4652    1.4124    1.3388

  Columns 7 through 11

    1.2985    1.1744    1.0551    0.9768    0.8989
    1.2864    1.1620    1.0540    0.9743    0.8913
    1.2802    1.1558    1.0517    0.9618    0.9002
    1.2811    1.1536    1.0516    0.9700    0.8885
    1.2682    1.1515    1.0487    0.9604    0.8891
    1.2682    1.1469    1.0561    0.9663    0.9008
    1.2626    1.1481    1.0568    0.9699    0.8865
    1.2628    1.1471    1.0469    0.9638    0.8877
    1.2611    1.1454    1.0460    0.9571    0.8944
    1.2443    1.1362    1.0466    0.9585    0.8944
    1.2443    1.1385    1.0452    0.9629    0.8944

% Gridshell curvature along y in [1/m]
ky =

  Columns 1 through 6

    3.2944    2.8356    2.3886    2.0182    1.7271    1.5137
    2.6608    2.5010    2.2053    1.9262    1.6869    1.4875
    2.2718    2.2608    2.0657    1.8666    1.6503    1.4623
    2.1344    2.0551    1.9819    1.8002    1.6273    1.4507
    2.5650    1.8622    1.8606    1.7479    1.5959    1.4392
    2.8483    1.7327    1.7614    1.6884    1.5649    1.4279
    2.9286    1.6285    1.6692    1.6290    1.5383    1.4191
    0.0150    1.5629    1.6214    1.5983    1.5046    1.4080
    0.1648    1.4736    1.5757    1.5677    1.5029    1.3968
    0.1405    1.3843    1.5224    1.5233    1.4647    1.3722
    0.1605    1.3430    1.4637    1.4950    1.4477    1.3461

  Columns 7 through 11

    1.3328    1.1965    1.0922    0.9932    0.9158
```

179	1.3191	1.1898	1.0808	0.9894	0.9135
180	1.3142	1.1747	1.0779	0.9866	0.9085
181	1.3004	1.1791	1.0756	0.9819	0.9099
182	1.2945	1.1761	1.0759	0.9928	0.9075
183	1.2989	1.1836	1.0789	0.9870	0.9089
184	1.2880	1.1745	1.0711	0.9882	0.9048
185	1.2779	1.1718	1.0809	0.9797	0.9162
186	1.2729	1.1787	1.0690	0.9798	0.9095
187	1.2713	1.1707	1.0668	0.9846	0.9095
188	1.2713	1.1708	1.0664	0.9812	0.9095

```matlab
% ############################################################
% Description
% ############################################################

% This function interfaces the Gridhsell analysis parameters to abaqus, runs the
% analysis, and retirieves the output.

% Author:
% Philippe Grönquist (pgroenquist@ethz.ch), August 2019


function Y = Gridshell_Analysis(X)

    % ####################################################################
    % Initialize
    % ####################################################################

    % Set current directory
    cd C:\Temp

    % Initialize, delete files from previous runs in case of error
    delete('GridshellInput.txt');
    delete('GridshellOutput.txt');
    delete('Gridshell.odb');
    delete('Gridshell.lck');

    % Rename values from input vector for clarity, as in "Gridshell_Setup.m"
    n  = X(1);
    L  = X(2);
    w  = X(3);
    h1 = X(4);
    h2 = X(5);


    % ####################################################################
    % Interface to Abaqus
    % ####################################################################

    % Write input for Abaqus
    data.cont= [n,...           % dataCont[0]
                L,...           % dataCont[1]
                w,...           % dataCont[2]
                h1,...          % dataCont[3]
                h2,...          % dataCont[4]
                ];

    % Define path of the necessary files
    path = 'C:\Temp';

    % Define name of the .txt input file
    name='GridshellInput.txt';

    % Call function to create txt interface file with input for python scripts
    CreateTxt(data, name, path);


    % ####################################################################
    % Run the Finite element analysis
    % ####################################################################

```

```
    % (Abaqus with user subroutines (UMAT) works only when opening matlab
    % from Intel Composer cmd!)

    % Option without Abaqus GUI
    ! abaqus cae noGUI=Gridshell_ParametricFEmodel.py

    % Option with Abaqus GUI
    %! abaqus cae script=Gridshell_ParametricFEmodel.py

    % Python script waits until job is completed
    disp('Analysis completed')


    % ######################################################################
    % Retrive output of FE analysis
    % ######################################################################

    % Initialize variables.
    filename = 'C:\Temp\GridshellOutput.txt';
    delimiter = ' ';
    startRow = 2;

    % Format for each line of text:
    %   column1: double (%f)
    %   column2: double (%f)
    %   column3: double (%f)
    %   column4: double (%f)
    formatSpec = '%f%f%f%f%[^\n\r]';

    % Open the text file.
    fileID = fopen(filename,'r');

    % Read columns of data according to the format.
    dataArray = textscan(fileID, formatSpec,'Delimiter', delimiter, 'MultipleDelimsAsOne', true, 'HeaderLines' ,startRow-1, 'ReturnOnError', false, 'EndOfLine', '\r\n');

    % Close the text file.
    fclose(fileID);

    % Create output variable
    Results = [dataArray{1:end-1}];


    % ######################################################################
    % Function output
    % ######################################################################
    Y = Results;

end


% ######################################################################
% Function: Generate the .txt interface file to Abaqus
% ######################################################################

function [] = CreateTxt(data, name, path)

    % Create file and open it
    fileName = fullfile(path,name);
```

```
    fileID = fopen(fileName,'w');

    % Header
    fprintf(fileID,'%s\n','Values to pass');
    fprintf(fileID,'\n');

    % Print variables
    fprintf(fileID,'%s\n','Continuous variables');
    fprintf(fileID,'%1.12e\n',data.cont);
    fprintf(fileID,'\n');

    % Print end
    fprintf(fileID,'%s','End');

    % Close file
    fclose(fileID);

end
```

```
% ##############################################################
% Description
% ##############################################################

% This function returns the curvatures along the mid-strips of the gridshell bilayer strips,
% calculated from their coordinate retrieved from FE model displacements.
% !! This function is custom to the number of strips chosen and needs to be
% adapted accordingly for a number of strips in the gridshell other than
% 6 !!

% Author:
% Philippe Grönquist (pgroenquist@ethz.ch), August 2019

function [Kx,Ky,Y] = Gridshell_Curvature(X,L)

% Description of input vector X
% Row 1: Reference Point number
% Row 2: X displacement
% Row 3: Y displacement
% Row 4: Z displacement

length          = L;
screwdistance   = length/4;


% ##############################################################
% Create matrix of strating coordinates
% ##############################################################

count = 1;
for i = 1:3
    for j = 1:3

    count = count + 2;

    RPy = screwdistance*j;
    RPx = screwdistance*i;

    RP(count,1)  = screwdistance*i;
    RP(count,2)  = screwdistance*j;

    RP(count+1,1)  = screwdistance*i;
    RP(count+1,2)  = screwdistance*j;

    end

end

RP(1:2,:) = [];

% add zeros as staring points
RP = [RP zeros(size(X,1),1)];


% ##############################################################
% Update with displacement values from FE model
% ##############################################################

% End coordinates of points = starting + displacement coords from FE
```

```
% analysis
A = RP(:,1) + X(:,2);
B = RP(:,2) + X(:,3);
C = RP(:,3) + X(:,4);


% ##############################################################
% X-direction
% ##############################################################

% select RP 4,10,16 and 8,10,12
% x-direction strip
P1 = [A(4) B(4) C(4)];
P2 = [A(10) B(10) C(10)];
P3 = [A(16) B(16) C(16)];

% distances between the 3 points
a = ((P2(1)-P1(1))^2 + (P2(2)-P1(2))^2 + (P2(3)-P1(3))^2)^(1/2);
b = ((P3(1)-P1(1))^2 + (P3(2)-P1(2))^2 + (P3(3)-P1(3))^2)^(1/2);
c = ((P3(1)-P2(1))^2 + (P3(2)-P2(2))^2 + (P3(3)-P2(3))^2)^(1/2);

% radius of circle in mm
Rx = (a*b*c)*(2*a^2*b^2 + 2*b^2*c^2 + 2*c^2*a^2 - a^4 - b^4 - c^4)^(-1/2);
Kx = 1/Rx;


% ##############################################################
% Y-direction
% ##############################################################

% y-direction strip
P4 = [A(8) B(8) C(8)];
P5 = [A(10) B(10) C(10)];
P6 = [A(12) B(12) C(12)];

% distances between the 3 points
a = ((P5(1)-P4(1))^2 + (P5(2)-P4(2))^2 + (P5(3)-P4(3))^2)^(1/2);
b = ((P6(1)-P4(1))^2 + (P6(2)-P4(2))^2 + (P6(3)-P4(3))^2)^(1/2);
c = ((P6(1)-P5(1))^2 + (P6(2)-P5(2))^2 + (P6(3)-P5(3))^2)^(1/2);

% radius of circle in mm
clear a b c
Ry = (a*b*c)*(2*a^2*b^2 + 2*b^2*c^2 + 2*c^2*a^2 - a^4 - b^4 - c^4)^(-1/2);
Ky = 1/Ry;


% ##############################################################
% Function output
% ##############################################################

% Gaussian curvature
Y = Kx*Ky;

end
```

```python
# #############################################################
# Description
# #############################################################

# This python script generates a parametric FE model of a beech wood self-shaping
gridshell in Abaqus 6.14.

# Authors:
# Prijanthy Panchadcharam, Mai 2018
# Falk Wittel, Mai 2018
# Philippe Grönquist (pgroenquist@ethz.ch), Mai 2018

# Abaqus default initialization
from part import *
from material import *
from section import *
from assembly import *
from step import *
from interaction import *
from load import *
from mesh import *
from optimization import *
from job import *
from sketch import *
from visualization import *
from connectorBehavior import *
from abaqus import *
from abaqusConstants import *

# Import for accessing the results (Abaqus specific)
import odbAccess

# Import for python
import sys
import os
import numpy as np
import math
from textRepr import *

# Name model
mdb.models.changeKey(fromName='Model-1', toName='Grid shell')
myModel=mdb.models['Grid shell']

##################################################################################
# Import input parameters from Matlab
##################################################################################

# Open file
path='C:\Temp'
name='GridshellInput.txt'
fileNameComplete = os.path.join(path, name)
file = open(fileNameComplete, 'r')

# Store information of all lines
lineList = file.read().split('\n')

# Close the file
file.close()

# Find headers
tableTitleLineIndex = []
for i,line in enumerate(lineList):
    if re.match('^Continuous', line) != None:
       tableTitleLineIndex.append(['Continuous',i])
    if re.match('^End', line) != None:
       tableTitleLineIndex.append(['End',i])

# Initialize the result variables
dataCont=[]

# Get Floats
for line in lineList[tableTitleLineIndex[1][1]+1:tableTitleLineIndex[2][1]-1]:
```

```python
    dataCont.append(float(line.split(',')[0]))


######################################################################################
# Parameters setup
######################################################################################
n      = int(dataCont[0]/2+1)                  # n-1 = number of screws on a strip
length = dataCont[1]                           # Strip length in mm
width  = dataCont[2]                           # Strip width in mm
passivethickness = dataCont[3]                 # Thickness of passive layer in mm
activethickness  = dataCont[4]                 # Thickness of active layer in mm
Thickness = passivethickness + activethickness
screwdistance = length/n                       # Distance between 2 screws
screwdiameter = 2.0                            # Diamater of screws
tempinf = 20                                   # Initial temperature
tempsup = 12                                   # End temperature
MeshSize = width/2                             # Mesh size in mm
Steptime = 1                                   # Step time unitless


######################################################################################
# Parts
######################################################################################

# Generate strips with holes (for screw connections)

# Active layer
myModel.ConstrainedSketch(name='__profile__', sheetSize=200.0)
myModel.sketches['__profile__'].rectangle(point1=(0.0, 0.0), point2=(length, width))

for i in range(1,n):
    myModel.sketches['__profile__'].CircleByCenterPerimeter(center=(i*screwdistance, width/2), point1=(i*screwdistance, width/2+screwdiameter/2))

myModel.Part(dimensionality=THREE_D, name='AL', type= DEFORMABLE_BODY)
myModel.parts['AL'].BaseSolidExtrude(depth=activethickness, sketch=myModel.sketches['__profile__'])

# Passive layer
myModel.ConstrainedSketch(name='__profile__', sheetSize=200.0)
myModel.sketches['__profile__'].rectangle(point1=(0.0, 0.0), point2=(length, width))

for j in range(1,n):
    myModel.sketches['__profile__'].CircleByCenterPerimeter(center=(j*screwdistance, width/2), point1=(j*screwdistance, width/2+screwdiameter/2))

myModel.Part(dimensionality=THREE_D, name='PL', type= DEFORMABLE_BODY)
myModel.parts['PL'].BaseSolidExtrude(depth=passivethickness, sketch=myModel.sketches['__profile__'])


######################################################################################
# Create sets & naming
######################################################################################

# Name surfaces to be tied later
topFace = myModel.parts['AL'].faces.findAt(((1,width/2,0),) )
botFace = myModel.parts['PL'].faces.findAt(((1,width/2,passivethickness),) )
topRegion = (topFace,)
botRegion = (botFace,)
myModel.parts['AL'].Surface(name='Surf-AL', side1Faces=topRegion)
myModel.parts['PL'].Surface(name='Surf-PL', side1Faces=botRegion)

# Modification: For surfaces instead of edges: Used for RP coupling to layers
for i in range(1,n):
    topedgeS = myModel.parts['AL'].faces.findAt(((i*screwdistance,width/2-screwdiameter/2,activethickness-0.1),) )
    botedgeS = myModel.parts['PL'].faces.findAt(((i*screwdistance,width/2-screwdiameter/2,0.1),) )
    topEdgesS = (topedgeS,)
```

```
    botEdgesS = (botedgeS,)
    myModel.parts['AL'].Surface(name='LA ' + str(i), side1Faces=topEdgesS)
    myModel.parts['PL'].Surface(name='LP ' + str(i), side1Faces=botEdgesS)

# Find and name parts, necessary for the supports
topedge1 = myModel.parts['PL'].vertices.findAt(((0,width,0),) )
botedge1 = myModel.parts['PL'].vertices.findAt(((0,0,0),) )
topedge2 = myModel.parts['PL'].vertices.findAt(((length,width,0),) )
botedge2 = myModel.parts['PL'].vertices.findAt(((length,0,0),) )
topEdges1 = (topedge1,)
botEdges1 = (botedge1,)
topEdges2 = (topedge2,)
botEdges2 = (botedge2,)
myModel.parts['PL'].Set(name='AUFLAGER 1', vertices=topEdges1)
myModel.parts['PL'].Set(name='AUFLAGER 2', vertices=botEdges1)
myModel.parts['PL'].Set(name='AUFLAGER 3', vertices=topEdges2)
myModel.parts['PL'].Set(name='AUFLAGER 4', vertices=botEdges2)

# Name surfaces, for surface contacts between vertical and horizontal strips
topSurface = myModel.parts['AL'].faces.findAt(((1,width/2,activethickness),) )
botSurface = myModel.parts['PL'].faces.findAt(((1,width/2,0),) )
topRegion = (topSurface,)
botRegion = (botSurface,)
myModel.parts['AL'].Surface(name='Surf-contact-AL', side1Faces=topRegion)
myModel.parts['PL'].Surface(name='Surf-contact-PL', side1Faces=botRegion)


######################################################################################
# Material
######################################################################################

# Define swelling coefficients
myModel.Material(name='Material-1')
myModel.materials['Material-1'].Expansion(table=((0.002, 0.004, 0.0001), ),
type=ORTHOTROPIC)

# Define moisture-dependent engineering constants between 0 and 30 % moisture content
myModel.materials['Material-1'].Elastic(table=((2505.9, 862.0,
    16854.3, 0.849703595, 0.055647712, 0.016761444, 652.61, 1466.74, 1082.28,
    1.0), (2446.2, 838.6, 16571.9, 0.849653412, 0.053960203, 0.016125284,
    637.42, 1451.48, 1064.56, 2.0), (2386.5, 815.2, 16289.5, 0.849748572,
    0.052278114, 0.015493129, 622.23, 1436.22, 1046.84, 3.0), (2326.8, 791.8,
    16007.1, 0.850001962, 0.050601733, 0.014865189, 607.04, 1420.96, 1029.12,
    4.0), (2267.1, 768.4, 15724.7, 0.850428037, 0.048931367, 0.014241692,
    591.85, 1405.7, 1011.4, 5.0), (2207.4, 745.0, 15442.3, 0.85104307,
    0.047267346, 0.013622882, 576.66, 1390.44, 993.68, 6.0), (2147.7, 721.6,
    15159.9, 0.851865442, 0.045610025, 0.01300902, 561.47, 1375.18, 975.96,
    7.0), (2088.0, 698.2, 14877.5, 0.852916001, 0.043959786, 0.012400389,
    546.28, 1359.92, 958.24, 8.0), (2028.3, 674.8, 14595.1, 0.854218485,
    0.042317038, 0.011797291, 531.09, 1344.66, 940.52, 9.0), (1968.6, 651.4,
    14312.7, 0.855800043, 0.040682227, 0.011200055, 515.9, 1329.4, 922.8,
    10.0), (1908.9, 628.0, 14030.3, 0.857691871, 0.03905583, 0.010609034,
    500.71, 1314.14, 905.08, 11.0), (1849.2, 604.6, 13747.9, 0.859929995,
    0.037438367, 0.010024612, 485.52, 1298.88, 887.36, 12.0), (1789.5, 581.2,
    13465.5, 0.862556242, 0.0358304, 0.009447203, 470.33, 1283.62, 869.64,
    13.0), (1729.8, 557.8, 13183.1, 0.865619458, 0.034232539, 0.008877259,
    455.14, 1268.36, 851.92, 14.0), (1670.1, 534.4, 12900.7, 0.869177043,
    0.032645447, 0.008315268, 439.95, 1253.1, 834.2, 15.0), (1610.4, 511.0,
    12618.3, 0.873296914, 0.031069848, 0.007761767, 424.76, 1237.84, 816.48,
    16.0), (1550.7, 487.6, 12335.9, 0.878060023, 0.02950653, 0.007217337,
    409.57, 1222.58, 798.76, 17.0), (1491.0, 464.2, 12053.5, 0.883563645,
    0.027956358, 0.006682616, 394.38, 1207.32, 781.04, 18.0), (1431.3, 440.8,
    11771.1, 0.889925711, 0.026420277, 0.006158303, 379.19, 1192.06, 763.32,
    19.0), (1371.6, 417.4, 11488.7, 0.897290599, 0.024899327, 0.005645165,
    364.0, 1176.8, 745.6, 20.0), (1311.9, 394.0, 11206.3, 0.905836983,
    0.023394651, 0.005144048, 348.81, 1161.54, 727.88, 21.0), (1252.2, 370.6,
    10923.9, 0.915788665, 0.021907511, 0.004655883, 333.62, 1146.28, 710.16,
    22.0), (1192.5, 347.2, 10641.5, 0.927429781, 0.020439303, 0.004181702,
    318.43, 1131.02, 692.44, 23.0), (1132.8, 323.8, 10359.1, 0.941126602,
    0.018991577, 0.003722648, 303.24, 1115.76, 674.72, 24.0), (1073.1, 300.4,
    10076.7, 0.957359521, 0.017566053, 0.003279993, 288.05, 1100.5, 657.0,
    25.0), (1013.4, 277.0, 9794.3, 0.976771261, 0.016164652, 0.002855156,
```

```
    272.86, 1085.24, 639.28, 26.0), (953.7, 253.6, 9511.9, 1.000241763,
    0.014789524, 0.002449724, 257.67, 1069.98, 621.56, 27.0), (894.0, 230.2,
    9229.5, 1.029008758, 0.013443079, 0.002065477, 242.48, 1054.72, 603.84,
    28.0), (834.3, 206.8, 8947.1, 1.064870182, 0.012128033, 0.001704423,
    227.29, 1039.46, 586.12, 29.0), (774.6, 183.4, 8664.7, 1.11054157,
    0.010847457, 0.001368827, 212.1, 1024.2, 568.4, 30.0)),
    temperatureDependency=ON, type=ENGINEERING_CONSTANTS)

myModel.HomogeneousSolidSection(material='Material-1', name= 'Section-1',
thickness=None)

# Assign material to strips
myModel.parts['AL'].SectionAssignment(offset=0.0, offsetField=  '',
offsetType=MIDDLE_SURFACE, region=  (myModel.parts['AL'].cells,),
sectionName='Section-1'  , thicknessAssignment=FROM_SECTION)
myModel.parts['PL'].SectionAssignment(offset=0.0, offsetField=   '',
offsetType=MIDDLE_SURFACE, region=  (myModel.parts['PL'].cells,),
sectionName='Section-1'   , thicknessAssignment=FROM_SECTION)


########################################################################################
# Define local coordinate system
########################################################################################

# Radial = x-direction; Tangential = y-direction; Longitudinal = z-direction;

# Active layer
myModel.parts['AL'].DatumCsysByThreePoints(coordSysType=  CARTESIAN,
name='AL_coordinate', origin=(0,width,activethickness), point1=
        (length,width,activethickness), point2=(0,width,0))
a=myModel.parts['AL'].datums.keys()
myModel.parts['AL'].MaterialOrientation(  additionalRotationField='',
additionalRotationType=ROTATION_NONE, angle=0.0  , axis=AXIS_3, fieldName='',
localCsys= myModel.parts['AL'].datums[a[-1]], orientationType=SYSTEM, region=
(myModel.parts['AL'].cells,), stackDirection=STACK_3)

# Passive layer
myModel.parts['PL'].DatumCsysByThreePoints(coordSysType=  CARTESIAN,
name='PL_coordinate', origin= (0,width,passivethickness), point1=
(0,0,passivethickness), point2=  (0,width,0))
a=myModel.parts['PL'].datums.keys()
myModel.parts['PL'].MaterialOrientation(   additionalRotationField='',
additionalRotationType=ROTATION_NONE, angle=0.0   , axis=AXIS_3, fieldName='',
localCsys=   myModel.parts['PL'].datums[a[-1]], orientationType=SYSTEM, region=
(myModel.parts['PL'].cells,), stackDirection=STACK_3)


########################################################################################
# Bilayer assembly
########################################################################################

# Displace active and passive layer strips to match
myModel.rootAssembly.DatumCsysByDefault(CARTESIAN)
myAssembly=myModel.rootAssembly
myAssembly.Instance(dependent=ON, name='AL-1', part=  myModel.parts['AL'])
myAssembly.Instance(dependent=ON, name='PL-1', part=  myModel.parts['PL'])
myAssembly.translate(instanceList=('PL-1', ),  vector=(0.0, 0.0, -passivethickness))


########################################################################################
# Step
########################################################################################


myModel.StaticStep(initialInc=0.01, maxNumInc=1000, minInc=  1e-09, name='Step-1',
previous='Initial', timePeriod=Steptime)
myModel.steps['Step-1'].setValues(nlgeom=ON)
myModel.fieldOutputRequests['F-Output-1'].setValues(variables=
    ('S', 'NE', 'U', 'TEMP'))


########################################################################################
```

```python
# BC (Temperature) (1)
###########################################################################################


# Set the amplitude of temperature
myModel.TabularAmplitude(data=((0.0, tempinf), (1.0, tempsup)), name= 'Amp-1',
smooth=SOLVER_DEFAULT, timeSpan=STEP)

# Assign temeperature to strips
myAssembly.Set(cells=myAssembly.instances['AL-1'].cells +
myAssembly.instances['PL-1'].cells , name = 'Set-1' )
myModel.Temperature(createStepName='Initial',
crossSectionDistribution=CONSTANT_THROUGH_THICKNESS, distributionType=  UNIFORM,
magnitudes=(tempinf, ), name='Predefined Field-1', region=  myAssembly.sets['Set-1'])
myModel.predefinedFields['Predefined Field-1'].setValuesInStep(   amplitude='Amp-1',
magnitudes=(1.0, ), stepName='Step-1')


###########################################################################################
# Gridshell assembly
###########################################################################################

# Multiply the strip and displace each by distance of screws
myAssembly.LinearInstancePattern(direction1=(1.0, 0.0, 0.0), direction2=(0.0, 1.0,
0.0), instanceList=('AL-1', 'PL-1'),  number1=2, number2=n-1, spacing1=length,
spacing2=screwdistance)

# Rotate half of the displaced strips by 90 degrees
myList=()
for i in range(1,n):
    myList=myList+('AL-1-lin-2-'+str(i),'PL-1-lin-2-'+str(i),)
myAssembly.rotate(angle=90.0, axisDirection=(0.0, 0.0,
-activethickness-passivethickness), axisPoint=(length, (n-2)*screwdistance+width,
activethickness), instanceList=myList)

# Displace the rotated strips such that holes match
myAssembly.translate(instanceList=myList, vector=(-screwdistance+width/2,
screwdistance - width/2, -activethickness-passivethickness))

# Tie the active and passive layers
# Two cases: For not using same nodes as slaves for tie and for later hinge
for key in myAssembly.instances.keys():
    if key.find("AL")>=0:
        if key.find("AL-1-lin-2")>=0:
            surf1=myAssembly.instances[key].surfaces['Surf-AL']
            key1="P"+key[1:]
            surf2=myAssembly.instances[key1].surfaces['Surf-PL']
            myModel.Tie(adjust=ON, slave= surf1, name=  'Constraint-'+key,
            positionToleranceMethod=COMPUTED, master=  surf2, thickness=ON,
            tieRotations=ON)
        else:
            surf1=myAssembly.instances[key].surfaces['Surf-AL']
            key1="P"+key[1:]
            surf2=myAssembly.instances[key1].surfaces['Surf-PL']
            myModel.Tie(adjust=ON, master= surf1, name=  'Constraint-'+key,
            positionToleranceMethod=COMPUTED, slave=  surf2, thickness=ON,
            tieRotations=ON)


###########################################################################################
# Connection of strips via hinged wires
###########################################################################################

# Define reference points necessary for wire connections
RPlist=[]
for  i in range(1,n):
    for j in range(0,n-1):
        point1,point2=(i*screwdistance,width/2+j*screwdistance,activethickness),
        (i*screwdistance,width/2+j*screwdistance,-2*passivethickness-activethickness)
        myAssembly.ReferencePoint(point=point1)
        myAssembly.ReferencePoint(point=point2)
        myKeys=myAssembly.referencePoints.keys()
```

```
        RPlist=RPlist+[myKeys[0:2]] # append an element in RPlist composed of the two first elements in myKeys

# Reference number, retrieve internal numbering, minimum is set to bottom
x = min(RPlist[0])

# Connect the reference points with edges of holes
for i in range(1,((n-1)**2)*2+1):

    myAssembly.Set(name='Set-RP-'+str(i),referencePoints=(myAssembly.referencePoints[x+i-1],))

# Connect reference points with even numbers
dc = n - 1
j = 1
for i in range(1,(n-1)**2+1):
    myModel.Coupling(controlPoint= myAssembly.sets['Set-RP-'+str(i*2)], couplingType= KINEMATIC, influenceRadius=WHOLE_SURFACE, localCsys=None, name=
    'Coupling-gerade RP '+str(i), surface=myAssembly.instances['PL-1-lin-2-'+str(j)].surfaces['LP '+str(dc)], u1=ON, u2=ON, u3=ON, ur1=ON, ur2=ON, ur3=ON)
    if dc > 1:
        dc = dc -1
    else:
        dc = dc + (n-2)
        j = j + 1

# Connect reference points with odd numbers
for i in range(1,n):
    myModel.Coupling(controlPoint= myAssembly.sets['Set-RP-'+str(1 + (n-1)*2*(i-1))], couplingType= DISTRIBUTING, influenceRadius=WHOLE_SURFACE, localCsys=None, name=
    'Coupling-ungereade RP 1'+str(i), surface=myAssembly.instances['AL-1'].surfaces['LA '+str(i)], u1=ON, u2=ON, u3=ON, ur1=ON, ur2=ON, ur3=ON)

dc = 1
for j in range(3,(n-1)*2,2):
    dc = dc + 1
    for i in range(1,n):
        myModel.Coupling(controlPoint= myAssembly.sets['Set-RP-'+str(j + (n-1)*2*(i-1))], couplingType= DISTRIBUTING, influenceRadius=WHOLE_SURFACE, localCsys=None, name=
        'Coupling-ungereade RP '+str(j)+ str(i), surface=myAssembly.instances['AL-1-lin-1-'+str(dc)].surfaces['LA '+str(i)], u1=ON, u2=ON, u3=ON, ur1=ON, ur2=ON, ur3=ON)

# Create wire connection
for i in range(0,(n-1)**2*2,2):
    myAssembly.WirePolyLine(mergeType=IMPRINT, meshable= OFF, points=((myAssembly.referencePoints[x+i], myAssembly.referencePoints[x+1+i]),))
    myAssembly.Set(edges= myAssembly.edges, name='Wire-'+str(i))

# Define the screw connection as a hinge
myModel.ConnectorSection(assembledType=HINGE, name='hinge')

# Assign hinge to wire connection and define a local hinge coordinate system
myAssembly.DatumCsysByThreePoints(coordSysType= CARTESIAN, name='hinge coordinate', origin= (screwdistance,width/2,activethickness), point1= (screwdistance,width/2,0), point2= (screwdistance,width/2-screwdiameter/2,activethickness))

# Last wire definition contains all previous wires
myAssembly.SectionAssignment(region= myAssembly.sets['Wire-'+str((n-1)**2*2-2)], sectionName='hinge')
a=myAssembly.datums.keys()
myAssembly.ConnectorOrientation( localCsys1= myAssembly.datums[a[-1]], region= myAssembly.allSets['Wire-'+str((n-1)**2*2-2)])


#####################################################################################
# Contact of surfaces
#####################################################################################
```

```
# Choose surfaces for contact
Asurfacecontact=[]
Akey=[]
for i in range(1,n):
    Asurfacecontact = Asurfacecontact+['Surf-contact-AL',]
    Akey = Akey + ['AL-1-lin-2-'+str(i),]

Psurfacecontact=[]
Pkey=[]
for i in range(2,n):
    Psurfacecontact = Psurfacecontact + ['Surf-contact-PL',]
    Pkey = Pkey + ['PL-1-lin-1-'+str(i),]

Pkey = Pkey + ['PL-1',]
Psurfacecontact = Psurfacecontact + ['Surf-contact-PL',]

# Define interaction of surfaces
myModel.ContactProperty('IntProp-1')
myModel.interactionProperties['IntProp-1'].NormalBehavior(allowSeparation=ON, constraintEnforcementMethod=DEFAULT, pressureOverclosure=HARD)

for i in range(0,n-1):
    for j in range(0,n-1):
        myModel.SurfaceToSurfaceContactStd(adjustMethod=NONE, clearanceRegion=None, createStepName='Step-1', datumAxis=None, initialClearance=OMIT, interactionProperty='IntProp-1',
            master=myAssembly.instances[Pkey[j]].surfaces[Psurfacecontact[j]], name='surface contact'+str(i)+str(j),
            slave=myAssembly.instances[Akey[i]].surfaces[Asurfacecontact[i]], sliding=FINITE, thickness=ON)


##########################################################################################
# BC (Temperature) (2)
##########################################################################################


# Assign temperature Boundary Condition (BC)
for i in range(2,n):
    myAssembly.Set(cells=myAssembly.instances['AL-1-lin-1-'+str(i)].cells + myAssembly.instances['PL-1-lin-1-'+str(i)].cells , name = 'Set-'+str(i) )

for i in range(1,n):
    myAssembly.Set(cells=myAssembly.instances['AL-1-lin-2-'+str(i)].cells + myAssembly.instances['PL-1-lin-2-'+str(i)].cells , name = 'Set-'+str((n-1)+i) )

for i in range(2,(n-1)*2+1):
    myModel.Temperature(createStepName='Initial', crossSectionDistribution=CONSTANT_THROUGH_THICKNESS, distributionType=  UNIFORM, magnitudes=(tempinf, ), name='Predefined Field-'+ str(i), region= myAssembly.sets['Set-'+str(i)])
    myModel.predefinedFields['Predefined Field-' + str(i)].setValuesInStep( amplitude='Amp-1', magnitudes=(1.0, ), stepName='Step-1')


##########################################################################################
# Restraints and supports
##########################################################################################

# Define 4 points for supports of the gridshell
p1 = (screwdistance-width/2,-screwdistance+width/2,-2*passivethickness-activethickness)
p2 = ((n-1)*screwdistance+width/2,-screwdistance+width/2,-2*passivethickness-activethickness)
p3 = (screwdistance-width/2,(n-1)*screwdistance+width/2,-2*passivethickness-activethickness)
p4 = ((n-1)*screwdistance+width/2,(n-1)*screwdistance+width/2,-2*passivethickness-activethi
```

```
ckness)
myAssembly.ReferencePoint(point=p1)
myAssembly.ReferencePoint(point=p2)
myAssembly.ReferencePoint(point=p3)
myAssembly.ReferencePoint(point=p4)
myKeys=myAssembly.referencePoints.keys()

y = max(myKeys)
for i in range(1,5):

    myAssembly.Set(name='Set-AUFLAGER-'+str(i),referencePoints=(myAssembly.referencePoints[y-i+1],))

# Connect the support points to the strips
for i in range(2,5,2):
    myModel.Coupling(controlPoint= myAssembly.sets['Set-AUFLAGER-'+str(i)], couplingType= KINEMATIC, influenceRadius=WHOLE_SURFACE, localCsys=None, name=
    'Coupling-Auflager RP '+str(i), surface= myAssembly.sets['PL-1-lin-2-1.AUFLAGER '+str(i)], u1=ON, u2=ON, u3=ON, ur1=ON, ur2=ON, ur3=ON)

for i in range(1,4,2):
    myModel.Coupling(controlPoint= myAssembly.sets['Set-AUFLAGER-'+str(i)], couplingType= KINEMATIC, influenceRadius=WHOLE_SURFACE, localCsys=None, name=
    'Coupling-Auflager RP '+str(i), surface= myAssembly.sets['PL-1-lin-2-'+str(n-1)+'.AUFLAGER '+str(i)], u1=ON, u2=ON, u3=ON, ur1=ON, ur2=ON, ur3=ON)

# 3 sliding supports (sliding in global x- and y-direction)
for i in range(1,4):
    myModel.DisplacementBC(amplitude=UNSET, createStepName= 'Step-1', distributionType=UNIFORM, fieldName='', fixed=OFF, localCsys=None, name='verschiebliches Auflager ' + str(i),
        region= myAssembly.sets['Set-AUFLAGER-'+str(i)], u1=UNSET, u2=UNSET, u3=0.0, ur1=UNSET, ur2=UNSET, ur3=UNSET)

# 1 fixed support
myModel.DisplacementBC(amplitude=UNSET, createStepName= 'Step-1', distributionType=UNIFORM, fieldName='', fixed=OFF, localCsys=None , name='unverschiebliches Auflager 1',
    region= myAssembly.sets['Set-AUFLAGER-4'], u1=0.0, u2=0.0, u3= 0.0, ur1=UNSET, ur2=UNSET, ur3=UNSET)


#######################################################################################
# Mesh
#######################################################################################

# Active layer
myModel.parts['AL'].seedPart(deviationFactor=0.1,  minSizeFactor=0.1, size=MeshSize)
myModel.parts['AL'].generateMesh()
myModel.parts['AL'].setElementType(elemTypes=(ElemType( elemCode=C3D20R, elemLibrary=STANDARD), ElemType(elemCode=C3D15, elemLibrary=STANDARD), ElemType(elemCode=C3D10, elemLibrary=STANDARD)), regions=(myModel.parts['AL'].cells, ))

# Passive layer
myModel.parts['PL'].seedPart(deviationFactor=0.1, minSizeFactor=0.1, size=MeshSize)
myModel.parts['PL'].generateMesh()
myModel.parts['PL'].setElementType(elemTypes=(ElemType( elemCode=C3D20R, elemLibrary=STANDARD), ElemType(elemCode=C3D15, elemLibrary=STANDARD), ElemType(elemCode=C3D10, elemLibrary=STANDARD)), regions=(myModel.parts['PL'].cells, ))


#######################################################################################
# Job
#######################################################################################

ODBname = 'Gridshell'
mdb.Job(model='Grid shell', name=ODBname)
mdb.jobs[ODBname].setValues(numCpus=12, numDomains=12, numGPUs=1)
mdb.jobs[ODBname].submit(consistencyChecking=ON)
```

```
mdb.jobs[ODBname].waitForCompletion()


######################################################################################
# Process ODB
######################################################################################

# Access results file (odb) and retrieve z-displacement at evaluation point
odbPath = ODBname + '.odb'

# open the odb
myOdb = odbAccess.openOdb(path=odbPath)

# choose the time frame (negative indexing -> gets last element)
frame = myOdb.steps['Step-1'].frames[-1]

# container for results
results = []

# choose location
for i in range(1,2*(n-1)**2+1): # if n=4, loops from 1 to 18

    # evaluation point
    PointName = 'SET-RP-' + str(i)
    EndPoint = myOdb.rootAssembly.nodeSets[PointName]

    # choose what field output
    dispField = frame.fieldOutputs['U']
    dispSubField = dispField.getSubset(region=EndPoint)

    # retrieve displacements
    dataX = dispSubField.values[0].data[0]
    dataY = dispSubField.values[0].data[1]
    dataZ = dispSubField.values[0].data[2]

    results.append([i,dataX,dataY,dataZ])

# Close current odb file
myOdb.close()


######################################################################################
# Write results to a .txt file
######################################################################################

f = open('GridshellOutput.txt','w')
f.write('RPi Xcoord Ycoord Zcoord\n')
for s in range(0,len(results)):
    f.write('%d %d %d %e\n' % (results[s][0],results[s][1],results[s][2],results[s][3]))
f.close()
```
